# Traffic-light labels could reduce population intakes of calories, total fat, saturated fat, and sodium

**DOI:** 10.1371/journal.pone.0171188

**Published:** 2017-02-09

**Authors:** Teri E. Emrich, Ying Qi, Wendy Y. Lou, Mary R. L’Abbe

**Affiliations:** 1 Department of Human Nutrition, University of Toronto, Toronto, Ontario, Canada; 2 Dalla Lana School of Public Health, University of Toronto, Toronto, Ontario, Canada; Duke University School of Medicine, UNITED STATES

## Abstract

Traffic-light labelling has been proposed as a public health intervention to improve the dietary intakes of consumers. OBJECTIVES: to model the potential impact of avoiding foods with red traffic lights on the label on the energy, total fat, saturated fat, sodium, and sugars intakes of Canadian adults. METHODS: Canadian adults aged 19 and older (n = 19,915) who responded to the Canadian Community Health Survey (CCHS), Cycle 2.2. The nutrient levels in foods consumed by Canadians in CCHS were profiled using the United Kingdom’s criteria for traffic light labelling. Whenever possible, foods assigned a red traffic light for one or more of the profiled nutrients were replaced with a similar food currently sold in Canada, with nutrient levels not assigned any red traffic lights. Average intakes of calories, total fat, saturated fat, sodium, and sugars under the traffic light scenario were compared with actual intakes of calories and these nutrients (baseline) reported in CCHS. RESULTS: Under the traffic light scenario, Canadian’s intake of energy, total fat, saturated fat, and sodium were significantly reduced compared to baseline; sugars intakes were not significantly reduced. Calorie intake was reduced by 5%, total fat 13%, saturated fat 14%, and sodium 6%. CONCLUSION: Governments and policy makers should consider the adoption of traffic light labelling as a population level intervention to improve dietary intakes and chronic disease risk.

## Introduction

**Fig 1:**
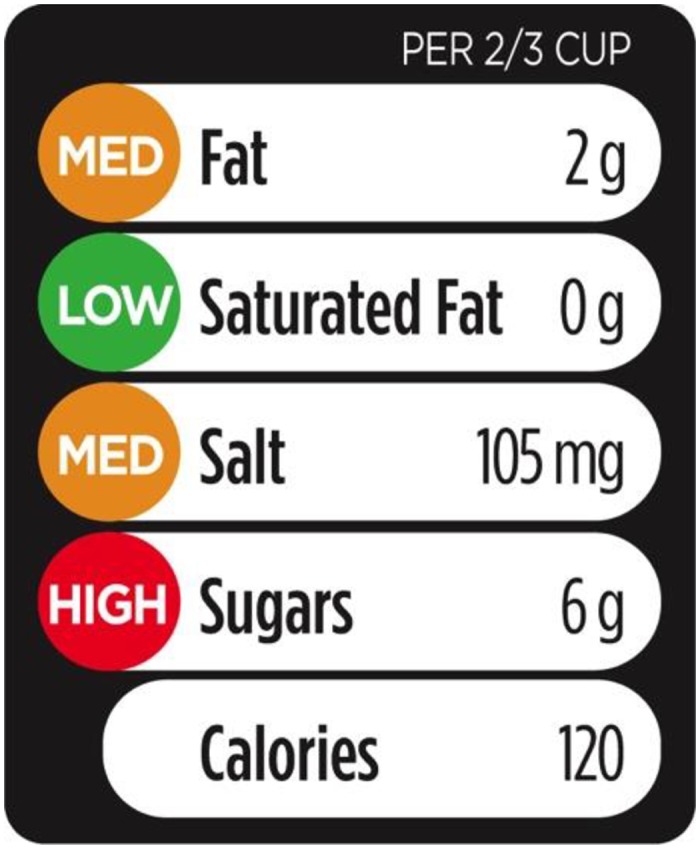
Example of a front-of-pack traffic light label.

Traffic-light labelling has been proposed as a public health intervention to reduce chronic disease risk by improving the dietary intakes of consumers [1,2]. Traffic-light labels are a form of interpretative front-of-pack (FOP) nutrition rating systems that provide information on the amount of calories and selected nutrients found within a specified amount of food (i.e. a nutrient-specific system) [3]. This particular type of system uses traffic-light colours to interpret for the consumer if the amounts of specific nutrients found in the product are “high”, “medium”, or “low” (Fig 1). A previous study identified traffic light labels as the FOP system that most consistently helped consumers to identify healthier choices [4]. Moreover, a modeling study carried out in Australia identified traffic-light labelling as a cost effective method for preventing obesity [5]. Research with Canadian consumers showed that they supported a single, standardized FOP system, with traffic-light labelling being their preferred choice [6].

Only a limited number of studies have examined the impact of traffic-light labelling on food purchases [5,7–10]. Interventions testing the impact of traffic-light labels on consumer food purchases suggest that consumers pursue a strategy of red avoidance in their food selections [8–10]. Thorndike et al [9] found that over the course of a cafeteria-based traffic-light labelling intervention, sales of items with red traffic-lights decreased 9.2% while sales of items with green traffic lights increased 4.5%. Similarly, Balcombe et al [8] found a strong aversion among consumers to market baskets that contained a mix of foods with any red traffic lights.

Beyond their potential impact on the food choices made by consumers, it has been suggested that FOP systems—such as traffic light labelling—may improve population nutrient intakes by stimulating manufacturers to reformulate their products in a more healthful way in order to meet the FOP system’s nutrient profiling criteria [3]. For instance, studies of summary indicator FOP systems, i.e.–systems that use a single symbol, icon, or score to provide summary information of the nutritional characteristics of food products [3], suggest that their underlying nutrient-profiles are in fact stringent enough to stimulate healthier product reformulations by manufacturers [11–14]. For example, Canadian manufacturer’s participating in the Health Check^™^ summary indicator system achieved a reduction of 80–150 mg of sodium in reformulated products in categories ranging from dinners and entrees to deli meats [14]. Similarly, with traffic light labelling, it is expected that manufacturers would reformulate their products to avoid the presence of ‘red’ traffic lights on their food labels.

Studies of the Choices International^™^ (http://www.choicesprogramme.org) summary indicator FOP system have found that population level intakes of energy, saturated and *trans* fat, sodium, and sugars could be reduced if usually consumed foods where replaced with foods that comply with the FOP systems criteria [15–17]. However, the potential impact of traffic light labelling on population level energy and nutrient intakes has not been studied. We hypothesize that if consumers avoided the consumption of foods which had red traffic lights for one or more nutrients in favour of similar foods with no red traffic lights, population level intakes of energy, total fat, saturated fat, sodium, and sugars would be reduced. The present study modeled the potential effect of avoiding foods with red traffic lights on the label and their replacement by similar products already sold in Canada (if available) on the energy, total fat, saturated fat, sodium, and sugars intakes of Canadian adults.

## Materials and methods

The energy, total fat, saturated fat, sodium, and sugars intake of Canadian adults (ages 19 and older) was calculated using data from the Canadian Community Health Survey, Cycle 2.2 Nutrition (CCHS 2.2). The CCHS 2.2 is a national, cross-sectional survey designed to provide reliable data on the food and nutrient intakes of Canadians [18]. The CCHS 2.2 included a sample of 35,107 individuals aged 0 and older who lived in private dwellings in Canada’s 10 provinces; excluded from the sample were members of the Canadian Forces and residents of Canada’s three territories, First Nation reserves or crown lands, institutions, and some remote areas. Data on food and nutrient intakes were collected through a 24-hour dietary recall, with a second recall collected for a sub-sample, using the United States Department of Agriculture’s Automated Multiple-Pass Method [19]. Complete details on the survey’s design, sample, and questionnaire have been reported on elsewhere [18,20]. This analysis focused on Canadian adults ages 19 and older and excluded pregnant and breastfeeding women, and individuals for whom food intake data was missing or incomplete. The final sample included 19,915 Canadians.

Red, amber and green colour coding was applied to all foods consumed by Canadians. Colour coding was determined using the criteria for food and drinks described in the United Kingdom’s *Guide to Creating a Front of Pack (FoP) Nutrition Label for Pre-packaged Products Sold Through Retail Outlets* (Table 1) [21]. The total fat, saturated fat, sodium, and sugars composition of each food and drink consumed was compared to the criteria. Food composition data for the CCHS 2.2 came from the Canadian Nutrient File (CNF), 2001b version, a recipe file and survey foods (items not in the CNF for which some nutritional information was available) [18]. The CNF is Canada’s standard reference food composition database. Most of the data is derived from the United States Department of Agriculture’s (USDA) Nutrient Database for Standard Reference [22]. However, the CNF includes Canadian specific fortification and regulatory standards and some foods consumed in Canada, not found in the USDA Nutrient Database for Standard Reference.

**Table 1:** Traffic light criteria for food and beverages [21].

	Green (Low)	Amber (Medium)	Red (High)
**Food (per 100 g)**			
**Fat (g)**	≤3.0	>3.0—≤17.5	>17.5
**Saturated Fat (g)**	≤1.5	>1.5—≤5.0	>5.0
**Salt (g)**	≤0.3	>0.3—≤1.5	>1.5
**Sugars**	≤5.0	>5.0—≤22.5	>22.5
**Beverages (per 100 ml)**			
**Fat (g)**	≤1.5	>1.5—≤8.75	>8.75
**Saturated Fat (g)**	≤0.75	>0.75—≤2.5	>2.5
**Salt (g)**	≤0.3	>0.3—≤0.75	>0.75
**Sugars**	≤2.5	>2.5—≤11.25	>11.25

Foods with red colour codes for one or more of the nutrient’s evaluated (total fat, saturated fat, sodium, and sugars) were replaced by similar foods that did not have red colour codes attributed to any of the nutrients evaluated. Replacement foods were existing comparable foods identified from the CNF, 2001b version supplemented with data from University of Toronto’s Food Label Information Program (FLIP 2010). FLIP is a database of branded foods sold in Canada with information on nutrient composition drawn from Canada’s mandatory nutrition label that has been described elsewhere [23]. Every effort was made to ensure the replacement food was as similar to the original as possible. Wherever possible, the original food was replaced by the same food of a different brand with a healthier nutrient possible. For example, a President’s Choice brand Muesli was replaced with am Alpen brand muesli. In some cases, the original raw ingredient or whole food was replaced by a version of the same food with a healthier nutrient profile. For example, lean ground beef was replaced with extra lean ground beef. Finally, in some instances some foods or recipes were replaced by the same food prepared slightly differently that yielded a healthier nutrient profile. For example, beef tenderloin prepared with the fat trimmed to 1/8 inch was replaced with beef tenderloin with fat trimmed to 0 inches. Table 2 provides further examples of substitutions made. Where it was not possible to find a comparable replacement food at these levels of similarity, the food was not replaced.

**Table 2:** Examples of foods of foods with one or more red traffic lights consumed by CCHS 2.2 participants and their no red traffic light replacements.

Original food consumed by CCHS 2.2 respondents	Replacement food
Babyfood, baked products, cookies, Arrowroot, Heinz	Selection: Arrowroot Cookies
Soup, chicken vegetable, canned, condensed	Campbell’s: Chicken vegetable (Canned) (Condensed unprepared)
Sweets, jams and preserves, apricot	Bonne Maman: Apricot Jam
Beef, ground, lean, raw	Beef, ground, extra lean, raw
Apricots, dried, sulphured, cooked, added sugar	Apricots, dried, sulphured, cooked without added sugar
Chicken, broiler, thigh, meat + skin, water chill raw	Chicken, broiler, thigh, meat, water chill, raw
Cereal, hot, oats, large flakes, dry, Rogers	Cereal, hot, pats, large flakes, dry, Quaker
Cracker, saltine, fat free, low salt	Compliments: Unsalted tops Soda Crackers
Sweets, pie fillings, canned, apple	E.D. Smith: Apple Pie Filling

### Analysis

Percentiles for usual nutrient intakes were estimated based on 24-hour two-day recalls. Software for Intake Distribution Estimation (SIDE) developed at Iowa State University (Version 1.11) was used for estimating the usual dietary nutrient intake distribution. Bootstrap replication method was used for the standard error estimation of all the estimates [24]. Baseline mean intakes of energy, total fat, saturated fat, sodium, and sugars were compared to the mean intakes under the traffic light scenario using a two-sample z-test. Data were weighted to represent the Canadian adult population. Statistical significant level was set at P<0.05. All calculations were carried out using SAS, version 9.3 (SAS Institute, Inc., Cary, NC).

### Ethics

The University of Toronto Health Sciences Research Ethics Board granted approval of this research (protocol reference #28017). As a secondary data analysis written informed consent for this research was not obtained from participants. This research followed Statistics Canada and the Statistics Canada Research Data Centre guidelines on data confidentiality. The data was anonymized and the research team did not have access to the personal identifiers of CCHS 2.2 participants.

## Results

**Fig 2:**
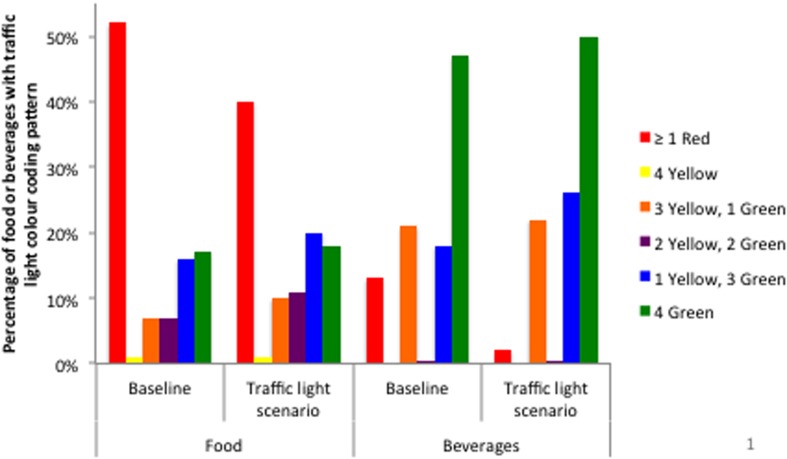
Traffic light labelling colour coding pattern of foods and beverages consumed by Canadian adults at baseline and under traffic light labelling scenario. Under the traffic light labelling scenario, whenever possible, foods that were reported as consumed by Canadians which met the criteria for at least one red colour code for one or more of the nutrients evaluated (total fat, saturated fat, sodium, and sugars) were replaced by similar foods that did not have red colour codes attributed to any of the nutrients evaluated.

In the CCHS 2.2, Canadians adults reported consuming 5655 unique foods and 495 unique beverages. At baseline, 52% of foods and 13% of beverages (weighted based on consumption) contained levels of one or more nutrients that qualified for a red traffic light (Fig 2). With respect to foods, the nutrient associated with the most red traffic lights at baseline was sodium (27% of foods) while sugars was associated with the least (14%) (Table 3). With respect to beverages, the nutrient associated with the most red traffic lights was sugars (10% of beverages) while sodium and fat were associated with the least (2%). Finding comparable products without any red traffic lights was not possible for all foods; however, under the traffic light scenario, the number of foods and beverages that qualified for at least one red traffic light dropped to 40% and 2%, respectively.

**Table 3:** Proportion of foods and beverages with red colour codes at baseline and under traffic light labelling scenario, by nutrient.

		Total fat	Saturated fat	Sodium	Sugars
**Foods (n = 5655)**	**Baseline**	**22%**	**23%**	**27%**	**14%**
	**Traffic light scenario**	**18%**	**19%**	**22%**	**10%**
**Beverages (n = 495)**	**Baseline**	**2%**	**3%**	**2%**	**10%**
	**Traffic light scenario**	**0%**	**0%**	**1%**	**1%**

Under the traffic light scenario, Canadian adults’ intake of energy, total fat, saturated fat, and sodium were significantly reduced compared to baseline (Table 4). Calorie intake was reduced by 5%, total fat by 13%, saturated fat by 14%, and sodium by 6% among Canadians 19 and over. The largest reductions were seen in men. Compared to baseline, men consumed 122 fewer calories, 12 g less total fat, 4 g less saturated fat, and 199 mg less sodium under the traffic light scenario (Table 4). Sugars intakes were not significantly reduced under the traffic light scenario compared to baseline.

**Table 4:** Mean energy and nutrient intake under the traffic light scenario compared with baseline.

		Energy (SE) (kcal/d)	Total fat (SE) (g/d)	Saturated fat (SE) (g/d)	Sodium (SE) (mg/d)	Sugars (SE) (g/d)
Daily Value*		2000	65	20	2400	-
**All**	**Baseline**	2065 (14)	75 (1)	25 (0)	3084 (27)	102 (1)
	**Traffic light scenario**	**1959 (13)**^a^	**65 (1)**^a^	**21 (0)**^a^	**2902 (26)**^a^	101 (1)
**Men**	**Baseline**	2382 (22)	87 (1)	28 (0)	3533 (46)	114 (2)
	**Traffic light scenario**	**2260 (21)**^a^	**75 (1)**^a^	**24 (0)**^a^	**3334 (45)**^a^	111 (2)
**Women**	**Baseline**	1750 (15)	64 (1)	21 (0)	2636 (29)	91 (1)
	**Traffic light scenario**	**1660 (14)**^a^	**55 (1)**^a^	**18 (0)**^a^	**2472 (27)**^a^	91 (1)

SE, standard error

*Daily Values are the reference standards upon which the calories and % Daily Value found on Canada’s Nutrition Facts table are based. The Daily Value for total fat is based on 30% of energy based on a 2000-Calorie diet. The Daily Value for saturated fat is based on a limit of 10% of energy. The Daily Value for sodium is 2400 mg. There is no current Daily Value for sugars. This reference value is based on the amount of a nutrient recommended to be consumed in the daily diet [25].

^a^Significantly lower than baseline intake (p<0.01).

Within Canada, nutrition labels are based on reference Daily Values for the amount of a nutrient recommended to be consumed in the daily diet by Canadians. Under the traffic light scenario, population level intakes of calories and total fat were reduced to at or below the recommended Daily Value. Women’s intake of saturated fat was also reduced to below the recommended Daily Value.

## Discussion

The results showed that traffic light labelling on food products could have a significant positive impact on the energy and nutrient intakes of Canadian adults by reducing their usual intakes of calories, total and saturated fat, and sodium. This is in line with the results of a different modeling study which projected that traffic light labelling would have a positive impact on calorie intake and body weight among Australians [5]. Moreover, these results are consistent with similar modeling studies conducted with summary indicator systems that showed that the selection of foods that met a certain nutritional standard over similar foods that did not meet these standards would result in positive changes in population level dietary intakes [15–17].

Within Canada, parents, consumers, and other stakeholders have called upon the Government to improve food labelling to help them control their sugars intakes [26]. In response, the Government of Canada proposed changes to the sugars information on food labels to make it easier to identify foods high in sugars [27]. While traffic light labelling could help consumers identify foods high in sugars, this study failed to demonstrate a potential improvement in dietary intakes of total sugars under a traffic light labelling scenario. It should be noted, however, that sugars was the nutrient to which the smallest proportion of red traffic lights were assigned at baseline (14%), with the majority of products already meeting the criteria for yellow and green traffic light labels. The lower number of red traffic lights for sugars prompted fewer substitution opportunities, likely explaining why no improvements were observed. If the sugars criteria were more stringent and, consequently, more products carried the red traffic light for this nutrient, more substitutions of lower sugars products would have been triggered. This suggests that in order to reduce population level intakes of sugars, traffic light labelling sugars criteria would need to be more stringent if it is to prompt the consumer to seek lower sugars options and the manufacturer to reformulate.

The present study assumed that when presented with two similar options, all consumers would select the option with a more desirable nutrient profile (i.e. with no red traffic lights), based on evidence suggesting that consumers pursue a strategy of red avoidance when using traffic light labels [8–10]. However, it is well documented that nutrition is not the only driver of food choice and is often secondary to other considerations such as taste and price [28–31]. Moreover, determinants of food choice include not only individual factors such as physiological and psychological factors, food preferences, nutrition knowledge, and perceptions of healthy eating, but also environmental factors that provide the context for individual behaviour [32]. Environmental factors determining food choice include things such as interpersonal relationships and the physical, economic, and social environments. Within this context, it cannot be assumed that consumers would automatically opt for foods with green traffic lights over foods with red ones. As a result, the optimal situation presented in this work likely overestimates the potential impact of traffic light labelling on Canadian adults’ nutrient intakes. It should be noted that under this modelling scenario and based on current choices available in Canadian market, comparable products without any red lights could not be found for 40% of foods and 2% of beverages. However as mentioned earlier, FOP systems can also act as a motivator for food companies to reformulate products to meet the threshold criteria; suggesting that the potential benefits could be underestimated if a substantial number of manufacturers reformulate to get below the red threshold criteria.

Given the expected impact of traffic light labelling on product reformulation by manufacturers, it is anticipated that if traffic light labelling were implemented in Canada the nutrient composition of the food supply would shift such that more products would be available without red traffic lights. Thus dietary intakes would improve without consumers consciously having to make more nutritious choices on the basis of labelling. However, some limitations to product reformulation should be noted. Nutrients such as sodium, fat, and sugars play functional roles in food in addition to providing flavour [33,34]. Sodium influences taste, texture, shelf life and food safety, while sugars impart sweetness mouthfeel/texture, bulk, colour, stability/preservation and acts as a fermentation substrate [34]. Reformulation must take into account all of these functions while producing a product that is still attractive to consumers [33–36]. To maintain consumer acceptance, reductions in nutrient such as sodium and sugars may need to be gradual and occur over several years [33].

Despite evidence of traffic light labelling’s potential to assist consumers in making healthier choices and improve dietary intakes, implementation of such a label is not presently under consideration in Canada. Since 2014, Health Canada has been in the process of reviewing Canada’s nutrition labelling regulations [25]; however, the proposed changes to the food label do not include the implementation of a front-of-pack traffic light label, or a front-of-pack label of any kind [27]. This despite the fact that a Health Canada consultation with consumers found that Canadians would like nutrition information on the food label to be more visual, “using a "green, yellow, red" system to indicate whether the quantity of nutrients is high, medium or low. [26]”

The results of this study demonstrate a positive impact of traffic light labelling on population level intakes of energy and nutrient intakes. Improvements were seen in intakes of calories, total and saturated fat, and sodium, but not sugars, when some of the foods with red traffic light labels were replaced with similar, currently available foods without red traffic light labels. The Canadian Government and policy makers should consider the adoption of traffic light labelling in their revision of Canada’s nutrition labelling regulations a population level intervention to improve dietary intakes and chronic disease risk.
